# Digital Approaches to Music-Making for People With Dementia in Response to the COVID-19 Pandemic: Current Practice and Recommendations

**DOI:** 10.3389/fpsyg.2021.625258

**Published:** 2021-04-23

**Authors:** Becky Dowson, Rebecca Atkinson, Julie Barnes, Clare Barone, Nick Cutts, Eleanor Donnebaum, Ming Hung Hsu, Irene Lo Coco, Gareth John, Grace Meadows, Angela O'Neill, Douglas Noble, Gabrielle Norman, Farai Pfende, Paul Quinn, Angela Warren, Catherine Watkins, Justine Schneider

**Affiliations:** ^1^Institute of Mental Health, University of Nottingham, Nottingham, United Kingdom; ^2^Blue Skies Singing Group Facilitator, Nottingham, United Kingdom; ^3^Chiltern Music Therapy, Chesham, United Kingdom; ^4^MHA, Derby, United Kingdom; ^5^OPUS Music CIC, Alfreton, United Kingdom; ^6^Cambridge Institute for Music Therapy Research, Anglia Ruskin University, Cambridge, United Kingdom; ^7^Music for Dementia, United Kingdom; ^8^Our Dementia Choir/Alzheimer's Society, Nottingham, United Kingdom; ^9^Live Music Now, London, United Kingdom; ^10^JoCo Learning and Development, Nottingham, United Kingdom; ^11^SongTime CIC, Nottingham, United Kingdom

**Keywords:** dementia, COVID-19, music, singing, technology, internet, digital arts, videoconferencing

## Abstract

Before COVID-19, dementia singing groups and choirs flourished, providing activity, cognitive stimulation, and social support for thousands of people with dementia in the UK. Interactive music provides one of the most effective psychosocial interventions for people with dementia; it can allay agitation and promote wellbeing. Since COVID-19 has halted the delivery of in-person musical activities, it is important for the welfare of people with dementia and their carers to investigate what alternatives to live music making exist, how these alternatives are delivered and how their accessibility can be expanded. This community case study examines recent practice in online music-making in response to COVID-19 restrictions for people with dementia and their supporters, focusing on a UK context. It documents current opportunities for digital music making, and assesses the barriers and facilitators to their delivery and accessibility. Online searches of video streaming sites and social media documented what music activities were available. Expert practitioners and providers collaborated on this study and supplied input about the sessions they had been delivering, the technological challenges and solutions they had found, and the responses of the participants. Recommendations for best practice were developed and refined in consultation with these collaborators. Over 50 examples of online music activities were identified. In addition to the challenges of digital inclusion and accessibility for some older people, delivering live music online has unique challenges due to audio latency and sound quality. It is necessary to adapt the session to the technology's limitations rather than expect to overcome these challenges. The recommendations highlight the importance of accessibility, digital safety and wellbeing of participants. They also suggest ways to optimize the quality of their musical experience. The pandemic has prompted innovative approaches to deliver activities and interventions in a digital format, and people with dementia and their carers have adapted rapidly. While online music is meeting a clear current need for social connection and cognitive stimulation, it also offers some advantages which remain relevant after COVID-19 restrictions are relaxed. The recommendations of this study are intended to be useful to musicians, dementia care practitioners, and researchers during the pandemic and beyond.

## Introduction

Musicians across the world have responded to the challenge of the COVID-19 pandemic with creative practice and innovative methods of delivery. To name just a few examples, lockdown choirs, courtyard serenades, live social media performances, and online music therapy sessions have sprung up to fill the void left by the cancellation of live musical performance and activities. In what can be seen as part of a “necessary digital revolution” in dementia care which has also been observed in medical settings (Cuffaro et al., 2020), the authors observed that many musical activities moved into an online format. This case study explores the forced changes brought about by digital delivery of musical activities for people living with dementia in the UK and examines the challenges this brings to both providers and participants. Firstly, we set out to collect available information on new modes of digital delivery, using a broad exploratory and descriptive approach to survey current practice within online music activities. Secondly, we narrowed our focus to online singing groups in order to describe and analyse current practice and understand the challenges involved in delivering this activity online. The rationale for this focus is that group singing is (in the UK) the most widespread and accessible type of live music activity specifically for people with dementia, and in order to develop the most relevant recommendations for practice it was germane to address this activity in particular. This case study aims to highlight promising initiatives, identify needs for further development, and make recommendations for providers and commissioners of dementia care. The findings may also be useful to those who are seeking to create new activities online, or to transform existing interventions into a virtual format.

## Context

Before Covid-19, dementia singing groups and choirs flourished, providing activity, cognitive stimulation and social support for thousands of people with dementia in the UK alone. Proof of popularity would be sufficient to explain their success, but evidence for the benefits of musical engagement for this population is also promising (McDermott et al., 2013; Sihvonen et al., 2017; van der Steen et al., 2017). In addition to the risk of virus spread through singing, a majority of people with dementia fit the category of older people advised to stay home for their own protection. The problem is that individuals with a diagnosis of dementia are likely to deteriorate through lack of the stimulation and the social contact that groups provide (Greenberg et al., 2020). Without the regular therapeutic opportunity of singing, their quality of life may decline and caring become more difficult, while both people with dementia and family carers miss out on the peer support that singing sessions often provide (Osman et al., 2014). Remote activities therefore offer an important medium for connection, relaxation and stimulation for people with dementia and their carers as they adjust to the risks of virus transmission. However, people with dementia and older people generally may be less likely than the wider population to have access to or engage with online activities. Therefore, the possibility of digital exclusion of some of the most isolated individuals must be taken into account (Nygård, 2008; Green and Rossall, 2018; Wallcook et al., 2019).

Digitally-mediated music making for people with dementia has arisen very rapidly in response to a specific crisis. Prior to COVID-19, use of telecommunications technology to deliver this kind of intervention was very limited; now, it is the primary means of delivery. This case study examines the adaptations made by UK musicians in the context of the pandemic.

### Aims

The speed of these developments means that there is very little existing literature or previous practice to draw upon. It is in this context that we proposed the following aims for this case study:

1. To document and categorise opportunities for singing and music-making that are currently available to people with dementia in the context of Covid-19 risk minimisation.
2. To describe the various features of these opportunities in practice, focusing on the technology used to deliver them, the approach of the facilitator, the adaptations which have been made and their overall accessibility.
3. To assess the barriers and facilitators to online singing in general, including the capacity and limitations of current technology.
4. To synthesise the findings from the various elements of this enquiry into a set of recommendations for online singing for people with dementia and their carers, in order to maximise its accessibility and benefits.

### Methods

There were two stages to this case study. In the first stage, relevant activities were documented and described, and in the second music practitioners reviewed the collated data and shared their own experiences. These stages are outlined below.

Stage 1: Author BD conducted online searches to find a range of music activities for people with dementia. The searches used the term “dementia” combined with either “singing” or “music.” The searches were conducted in English only. The activities described here were found using the following resources:

- List of virtual music activities on the Musical Map for Dementia on the “Music for Dementia” website (https://musicfordementia.org.uk/musicalmap/)
- Twitter search for relevant hashtags: #singing, #music, #dementia etc. (www.twitter.com)
- YouTube search for music, singing, dementia (www.youtube.com)
- Facebook search for music, singing, dementia (www.facebook.com)

The “Music for Dementia” website was selected as the basis for searches because of the previously-mentioned UK focus for this study. This website provides the most comprehensive overview of musical activities for people with dementia taking place around the UK. The search was not exhaustive as its aim was to uncover the most widely-used methods of delivering singing using digital methods and document how they are used, rather than to record every single example of this type of practice. From the results of these searches, a document collating the data we had retrieved was produced outlining the types of online sessions, the various platforms and some of the technological challenges and solutions. The initial categorisation of the data were undertaken by BD and reviewed by JS. The categories were determined by reading the descriptions of sessions provided, and by watching samples of the sessions if available.

Stage 2: The collated data on musical activities were circulated to the co-authors, all of whom were involved in relevant practice at the time of the study. The co-authors consisted of:

- professional musicians who lead online music sessions for people with dementia,
- music therapists with experiences of clinical work online with people with dementia
- people in leadership roles within organisations involved in providing or signposting online musical activities for people with dementia.

Table 1 gives the breakdown of the professions of the co-authors who contributed to the paper. Co-authors were identified through professional connections of JS and BD, known to be leaders in their field, some of whom suggested other co-authors whom we invited to contribute.

**Table 1:** Professions of co-authors involved in the study.

Description of profession*	**Number of co-authors**
Music therapist	7
Community musician	3
Singing group leader	4
Leaders in providing/signposting organisations	2

* *In some cases, co-authors could be categorised in more than one of these professional descriptions. In these cases, they were classified as the profession best representing the capacity in which they contributed to enquiry*.

These co-authors provided feedback and shared their personal experiences, challenges and discoveries from several months of involvement in virtual music provision. The process for the development of the paper is outlined below:

1. Firstly, collated data on music activities were shared. This contained initial results of the searches and reflections on some of the challenges of online delivery and their possible solutions.
2. Co-authors responded with comments and suggestions about this document, as well as reflections on their own practice and experiences. Not all of the co-authors were exclusively involved in delivering online singing, but their experience with other online music activities has much in common with online singing and is therefore highly relevant to this enquiry. Some co-author feedback was delivered in written form and some recorded in notes written by BD during online discussions. All this material was integrated into the collated data and used by BD and JS to develop recommendations for online singing.
3. The prototype recommendations were circulated to all co-authors for comments, and co-authors attended a video call to review and refine the recommendations (This led to the removal of some very specific pieces of technological advice which might not remain applicable in the future, and the addition of the recommendations about self-care and sustainable practice).

We investigated the main issues affecting the viability of online alternatives to singing face to face. Finally, we listed and refined the features that appear to differentiate successful implementation of online singing from less successful attempts according to the experiences of the co-authors in their own practice. Having reached a point of agreement between all co-authors with no new information being introduced by them, we formulated these as recommendations for best practice. Our approach to integrating comments and suggestions from co-authors and refining the recommendations was informed by consensus methodologies (Fink et al., 1984), but this was primarily a pragmatic enquiry rather than an exhaustive survey, intended to capture recent developments in a new and rapidly changing situation.

## Details

We identified nearly 50 examples of online musical interventions, provided by individuals, groups and organisations based in the UK. The following section outlines the types of sessions, activities and platforms which are currently in use. The intention behind collecting this data was to describe the context for the case study by painting a broad picture of the state of play mid-pandemic in the UK and to underpin the expert opinions which were sought in the second part of the enquiry.

### Types of Sessions

Our search found that the kinds of sessions being offered usually fall into one of five categories:

**Live fully-interactive sessions**—participants attend the session through a video calling platform and interact with each other and the facilitator in real time. The participants and the facilitator can all see each other and communicate.

**Live semi-interactive sessions**—participants watch the session as it is broadcast live by the facilitator. They cannot see the other participants, but some interaction is possible e.g., through typed chat.

**Live non-interactive sessions**—participants watch the sessions as it is broadcast live, but there is no interaction between the participants and the facilitator.

**Pre-recorded sessions**—the session is recorded in advance and can be watched at any time (this could be a recording of a previously live session).

**Carer-facilitated session**—the session is led by a face to face companion, such as a carer, using printed or recorded instructions or resources supplied online by the provider.

### Types of Activities

**Figure 1:**
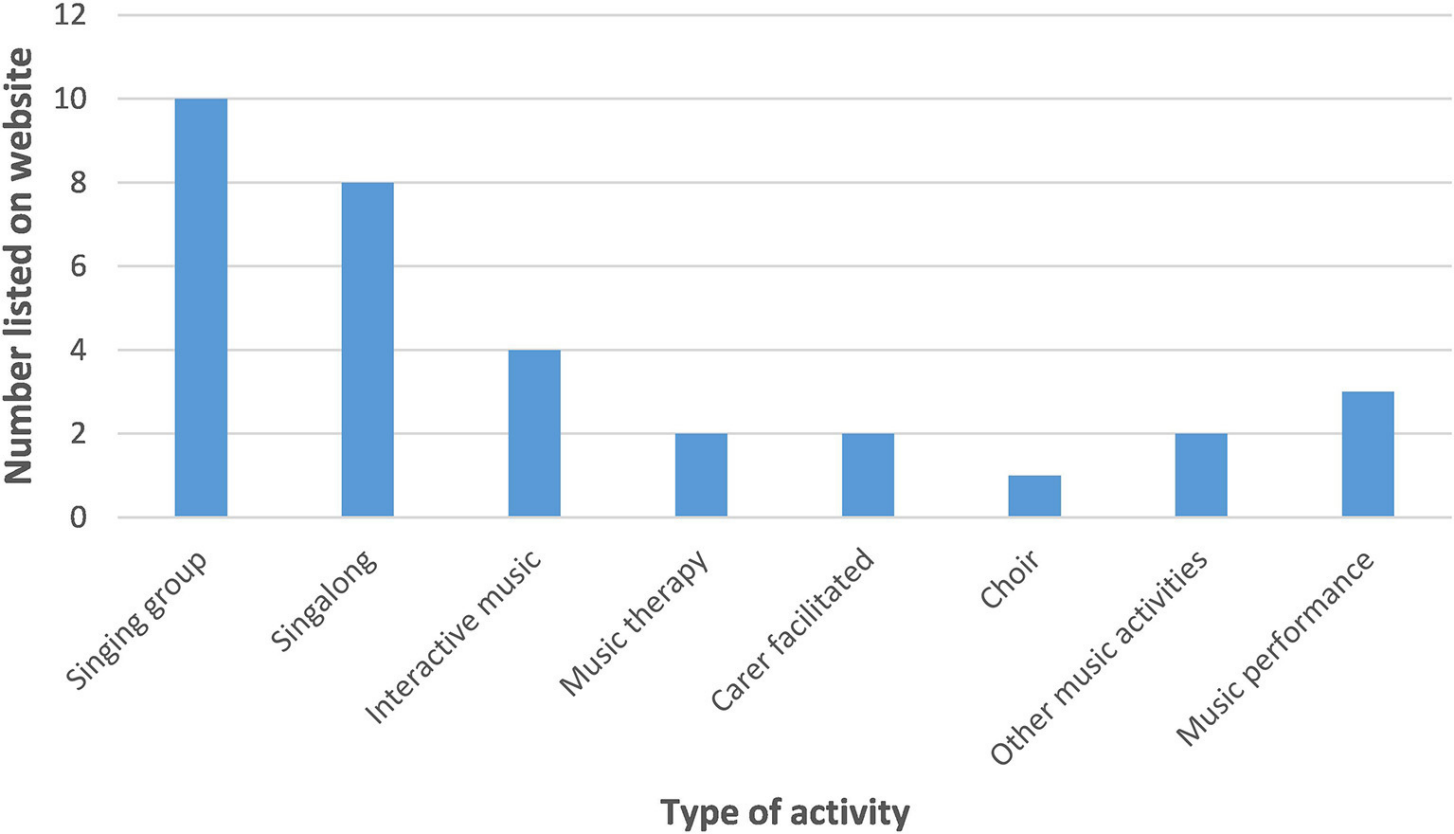
Online music activities for people with dementia listed on Music for Dementia website (July 2020).

The types of activities found during the search are outlined in Table 2, along with some examples. The description column contains a summary of each type of activity generated by the co-authors, while the examples are taken verbatim from the written descriptions of activities as they appear either on the Music for Dementia website or another hosting platform (e.g., the description in a YouTube video). A graphical breakdown of the types of activities listed on the Music for Dementia website is presented in Figure 1.

**Table 2:** Types of online music making activities.

**Type of activity**	**Description**	**Example**
Choir	The session is conducted as a choir rehearsal, adapted for the online format. The activities may include warm up, working on pieces and singing them through, with or without backing track or accompaniment. The participants will normally remain muted for most of the session, unless they need to speak.	“*We weren't sure how the singers would take to Zoom, but each week more faces are appearing, so that now over three-quarters of our caregiver-dementia pairs are participating in the virtual choir. They are inviting friends and relatives from around the world to join in — something that could not happen in real life. […] We've now added other activities on Zoom aimed at replicating the conversation and swapping of stories that happens naturally when people get together.” [Voices in Motion intergenerational choir]*
Singing group session	The session is run as a singing group, based around singing familiar songs which may be selected by the facilitator or the participants. Other activities such as warm ups, physical and vocal exercises, singing games and rounds may be included. Differs from a choir rehearsal in that it is less focused on working on and improving repertoire.	“*Regular members of our Music for the Mind and other community groups are being invited to join in at home via the magic of Zoom online sessions. It's lots of fun, a great way to keep up with singing or exercising together - and to see some familiar friendly faces. The sessions are held at the same time of the week that the group would normally meet.” [Music for the Mind]*
Singalong session	A selection of songs are presented for the participants to sing along to. These may be recorded as one continuous video or individual ones. Lyrics may be provided as subtitles.	“*This is a resource for anyone at home with dementia who might like a bit of a singalong. I will add more videos to this collection as the weeks and months go by. Each session has the timings of the songs in the description so you can skip to your favourites (and/or avoid the really bad ones!).” [My Mate George Online]*
Interactive music	An interactive online session which includes music activities but the focus is not on singing, although singing may be included.	“*A video series for people living with dementia. Play, sing or simply listen-along with me as I take popular tunes on a trip through a tapestry of sounds to promote wellbeing and happiness. New song-journeys added weekly.” [Song Journeys for Wellbeing by Engage and Immerse]*
Music therapy	Face to face live music therapy delivered by qualified music therapists using video calling technology. Sessions could involve singing, talking, playing music, listening to music, improvising and other musical activities. These would tend to be 1-to-1 sessions or small groups.	“*Together in Sound music therapy groups aim to bring the power of music to those living with dementia. … the social distancing measures and lockdown came into force halfway through our planned 10 week block. We have, however, been able to continue to deliver sessions online, and maintaining the community, fellowship, and music making that has been such an important part of Fridays at Saffron Hall … “ [Together in Sound, Anglia Ruskin University/Saffron Hall]*
Music performance	Live or recorded musical performance, possibly with activities included.	“*City of London Sinfonia will be offering a series of online concerts for you to enjoy from the comfort of your home. Each concert will feature a single CLS musician, broadcasting from their home, playing music and suggesting activity to do alongside their performance – drawing, making up stories, simple movement, mindfulness, etc.” [City of London Sinfonia] “Live Music Now have created LMNOnline, a free library of over 30 pre-recorded concerts encompassing a range of music genres made especially for people living and working in care homes for older people The resource is accompanied by guidance to support engagement and use.”[Live Music Now]*
Other activities involving music/singing	Any other form of activity where music may be included but it is not necessarily the main focus, or may not be included in every session. This could include online religious services.	“*At Care Visions Healthy Ageing, we understand the challenges of people living with dementia and their families. Our engaging and stimulating online video sessions support the needs of people at all stages of dementia and they are free! Whether you are near or far from your loved ones our 20-minute video sessions will help you share the contact experience.” [Care Visions Healthy Ageing]*
Carer-facilitated sessions	Recorded or written material which provides specific training or guidance for a carer to implement the session in their own time.	“*The programme works to up-skill carers and care staff, to enable them to use our tool to run effective and interactive music sessions with confidence. In doing so the programme offers innovative flexibility and cost-effectiveness to care homes, as they can use the tool as frequently as they need and whenever they choose.” [WellSinging by SoundUp]*

### Types of Platforms

**Figure 2:**
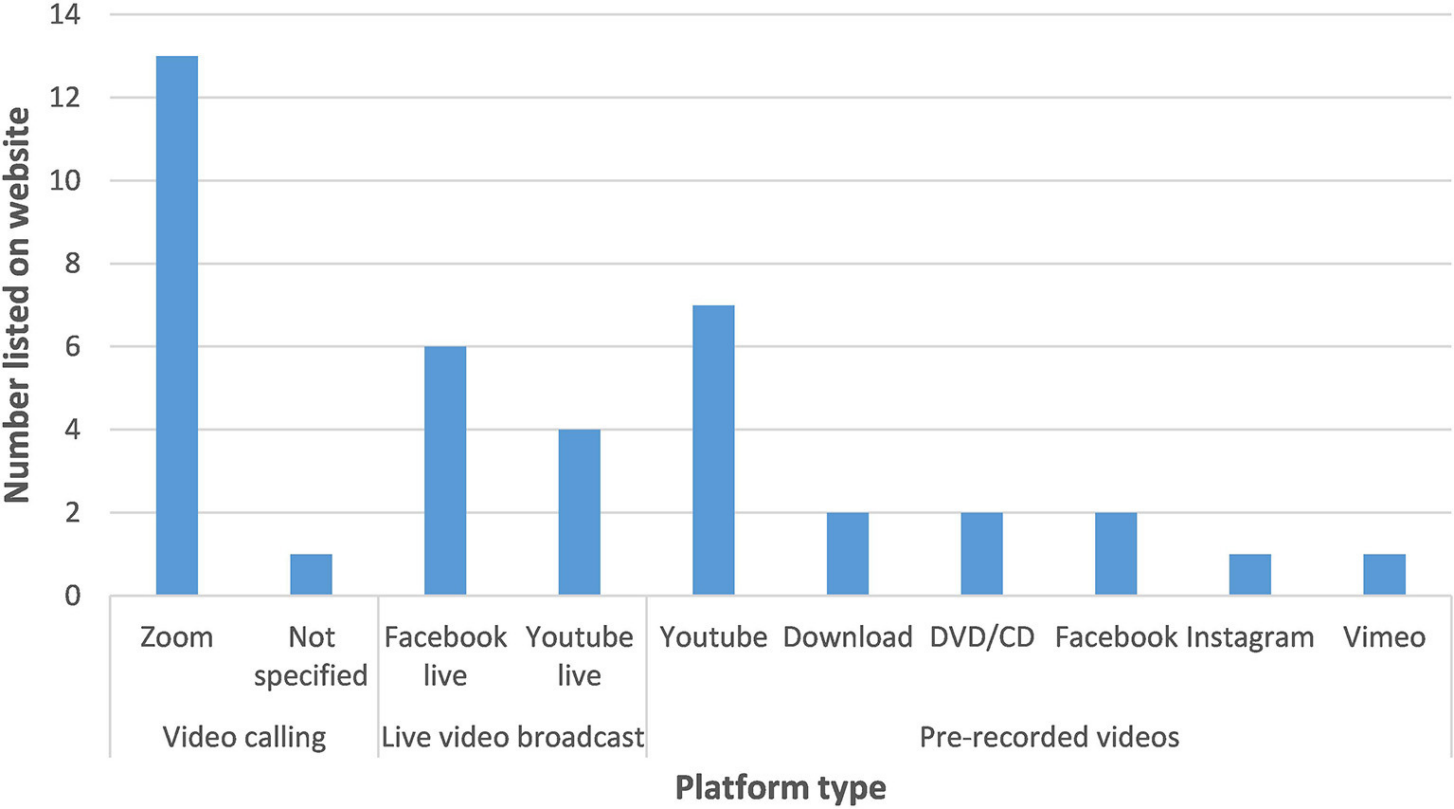
Platforms used to deliver music activities for people with dementia listed on Music for Dementia website (July 2020).

Table 3 summarises the online platforms which we found were used to share online music activities, while Figure 2 gives a graphical breakdown of the platforms used by activities listed on the Music for Dementia website (note that in some instances activities are offered on more than one platform). Overwhelmingly, Zoom (Zoom Video Communications) was the most popular choice for video calling, Facebook Live (Facebook Inc.) was commonly used for broadcasts, and video uploads were generally *via* YouTube (Google LLC). These choices probably reflect the availability and familiarity of these platforms. It was not uncommon for providers to offer sessions on several different platforms; for example, a live Zoom singing group alongside pre-recorded YouTube videos. In this way, providers could maximise the accessibility of the sessions whilst meeting different users' needs. The functionality of a platform often varies depending on whether the lead facilitator pays to use it or operates a free version.

**Table 3:** Digital platforms used for music provision.

**Platform**	**Examples**	**Device compatibility**					**Notes**
		**Smartphone Phone**	**Tablet**	**Computer**	**Telephone^**a**^**	**Smart TV**	
Video calling	Zoom	x	x	x	x	x	100 participants max, 40 min duration with free account. Can run from browser on computer.
	Google Meet	x	x	x	x	x	100 participants max, 60 min duration with free account. Phone-in only works with G-suite account. Can run from browser on computer.
	Skype	x	x	x			50 participants max, 4 h per call. Can run from browser on computer.
	FaceTime	x	x	x			32 user maximum, no time limit. Only works on Apple devices.
	WhatsApp	x					8 people max in a video call. Callers will see each other's phone numbers. Only works on smartphones.
Live video broadcast	Facebook Live	x	x	x		x	Live videos are saved and can be watched later. Broadcasts can be public or shared privately in a group. Participants can interact and respond with comments and emojis.
	Youtube Live	x	x	x		x	Live videos are saved and can be watched later. Broadcasts can be public or shared privately. Participants can interact and respond with comments.
Video streaming	Facebook	x	x	x		x	Videos can be shared publicly or privately in a group. Viewers can leave comments.
	Youtube	x	x	x		x	Videos can be shared publicly or privately (e.g., only those with the link can view). Viewers can leave comments.
	Vimeo	x	x	x		x	Videos can be shared publicly or privately (private sharing only with paid account). Viewers can leave comments.

a *Users without internet access can join video calls by dialling in from a mobile or landline telephone*.

## Discussion: Issues Affecting Viability of Online Singing

A number of issues were revealed in our enquiry; both challenges and related solutions. Here we consider first the technological issues affecting almost all users of digital music interventions, and then we look at issues which vary from person to person and can affect individual digital inclusion. Each issue which was identified by co-authors is described, with references to literature where relevant. We then address possible solutions and/or mitigations for these challenges.

### Technological Challenges and Solutions

The need to adapt existing software to meet the demands of online music activities has highlighted several limitations of the technology, as well as enabling the music-making community to devise some solutions. Connectivity, latency and variable sound quality emerged as the major issues in this respect.

#### Internet Connectivity

Co-authors reported that some participants who had access to the internet found that their connections were not fast or reliable enough to support video calling. Furthermore, even when a connectivity problem has a straightforward solution (e.g., restarting a WiFi router), a certain level of knowledge and confidence with technology is required. Although Office for National Statistics (ONS) data reports that 93% of UK households had access to the internet in 2019, connection speeds vary, with those in urban areas experiencing speeds which are on average almost twice those in rural areas (Office for National Statistics, 2019). Local disruptions to coverage may cause disappointment and frustration. Internet access in residential care homes may also be an issue in some cases, with 20% of UK care home workers reporting that their homes have no WiFi which is accessible to residents (Learner, 2019).

Co-authors discussed potential solutions to this issue, whilst acknowledging that it was often difficult to remotely solve participants' hardware issues especially during a session. Some WiFi users who are experiencing problems with their connection may be able to solve these by upgrading to a newer router, which in many cases is available for free on request from their internet service provider. Users who are experiencing slow connection speeds *via* WiFi may be able to speed up their connection by connecting their device directly to the router using an Ethernet cable if this option is available. For participants whose connection speeds preclude the use of live video streaming, audio-only calls or pre-recorded videos which can be downloaded in advance may be preferable. For care homes where WiFi is not available, connecting through a mobile internet “dongle” or providing a smartphone or tablet with a data-heavy SIM plan may provide a solution for individuals wishing to get online, although these are not without a cost.

#### Latency

Latency refers to the time delay between when an audio or video signal enters a system and when it emerges. All forms of telephone and internet calling introduce elements of latency, but the length of the time delay may vary between platforms and depends on the user's internet connection as well as the quality of their hardware. Some video conferencing platforms promise lower latency than others, but the consensus amongst the co-authors is that no currently available video conferencing platform has sufficiently low latency to make live large-group simultaneous music-making a viable choice. A typical video call latency of 100 milliseconds is not particularly noticeable when the call only involves speech, but makes simultaneously singing along to a song extremely difficult, because (if unmuted) the singers will hear each other asynchronously, which is very off-putting, especially since multiple participants will experience different amounts of latency. Upgrading hardware to minimise latency may be prohibitively expensive for musicians and participants alike.

Co-authors were in agreement that at present there is no solution to the issue of latency in the context of online community music making, and so the format of the sessions must be adapted. There are two possible options: adapt the session using the features of the technology, or adapt the type of music making. A very common implementation of the first option involves all of the participants apart from the facilitator muting their microphones. They are then able to join in with the music provided by the facilitator without latency issues, but the sense of collaborative group music making is somewhat lost. Co-authors had commonly used this approach. The second option involves musical adaptations: moving away from a musical style which is regulated by strict rhythm or pulse, toward a free, arrhythmic sonic experience which could be characterised as a soundscape. For example, June Boyce-Tillman has described creating a “Zoom Choir for Peace” based around a single shared drone note, and a set of chants that are based on the same chord (Boyce-Tillman, 2020). An alternative is to use a different platform; one co-author (a music therapist) found that some clients preferred to sing over the phone. Although it was a strange experience at first, it had more therapeutic potential, with the added advantage that phone calls have negligible latency. This co-author found that for some clients, engaging in phone singing built confidence and led to them being able to join a Zoom group after a few weeks.

#### Sound Quality

Co-authors discussed methods for optimizing sound quality of their sessions. Sound quality may be affected by both hardware and software issues. The quality of in-built device microphones is variable, and some video conferencing software is specifically designed to filter out background noise and prioritise speech, meaning that live music is sometimes detected as “background noise.” Furthermore, some software detects which participant is speaking and automatically filters out everyone else, making collaborative music-making difficult.

Several co-authors already owned or had invested in devices such as USB microphones and audio interfaces to improve their sound quality. Co-authors were also aware of less well-known features that can be used to improve the transmission of music. For instance, Zoom has an option to “Turn on Original Sound” which overrides the audio filtering mechanism. This means that the sound others hear is the sound received by the user's microphone. Co-authors had experimented with different ways of providing instrumental accompaniment for online sessions, including pre-recorded backing tracks which required prior testing to get the correct balance between accompaniment and voice.

### Challenges and Solutions to Digital Inclusion

The co-authors recognise that digital communications over the internet are unfamiliar to a sizeable proportion of the population. Not only is there a skills gap which disproportionately affects older people, but cost can be a barrier. For some people (not only those with memory problems) face to face training may be necessary. Concerns about data security may also be a deterrent to using online platforms. The acceptability of using an online intervention depends on an individual's personal situation, past experiences and understanding of the technological landscape to some extent. Fears and prejudices need to be addressed thoughtfully and some may prove insuperable.

#### Accessibility

It was the experience of most co-authors who had moved their existing music practice online that some of their clients were unable to join in either because they did not have access to the internet, or because they did not know how to use a specific required application. A sizeable minority of older people may not use the internet; data collated by AgeUK in 2018 indicated that 56% of people aged 75 and over had not used the internet in the past three months, while 36% of people aged 65 and over are lapsed or “never”-users (Green and Rossall, 2018). Among over 65s, the most common reason given for not having internet access at home is that it is not needed, with “lack of skills” cited as the next most common reason.

The co-authors recognise that a majority of over-65s are internet users, and a minority will never wish to use the internet. However, a sizeable proportion of this group may want to get online (or get back online in the case of lapsed users). We believe that it is likely that the COVID-19 pandemic has motivated increased internet use among older people, although evidence to support this speculation is not yet available. For those who wish to use the internet but need assistance, charities such as AgeUK offer computer classes (though ironically these may only be available online due to COVID-19). Also in the UK, the charity Abilitynet^1^ offers individualised support from vetted volunteers (many retired IT professionals) to help individuals overcome the barriers to digital technology use. Intergenerational engagement could also be employed to improve digital literacy; for instance, grandchildren supporting their grandparents to get online. Co-authors agreed that it was very important that providers did not assume that their participants would know how to get online, and suggested that detailed, clear and jargon-free instructions for joining video calls or downloading/streaming videos would be helpful. We also explored options to avoid excluding those who do not have internet access. For instance, some video conferencing platforms allow participants to join by phone. Alternatively, DVDs/CDs and activity packs could be supplied to those without internet, and one to one singing over the phone is another option.

#### Digital Security and Privacy

Some co-authors had encountered concerns about digital security and privacy in their online work. An Age UK survey found that 7% of respondents cited “privacy or security concerns” as a reason why they did not have internet at home. Older people may be under increased threat from cybercrime because of real or perceived vulnerability (Green and Rossall, 2018). The feeling of joining a new activity from one's own home could be anxiety-provoking for some, and this may be especially pronounced for a music therapy session, where the client may feel that confidentiality is less assured through a digital medium.

We understand that there is no easy solution to concerns about privacy and security online. Co-authors discussed ways in which practitioners could protect their participants' privacy, including using BCC when sending group emails to avoid sharing email addresses, and manually changing Zoom names to display first name only. Participants may also be reassured by the knowledge that the platform they are using for their musical activities is encrypted, and/or can only be joined by those with the specific link or password. Information and resources provided by charities such as Get Safe Online may help people to feel more secure and able to protect themselves from scams. Co-authors felt that security should not be downplayed, there is evidence of a rapid increase in phishing in recent months and COVID-19 related deceptions are rife; practitioners were alert to the very real risk of scams targeting participants.

#### Financial Barriers

Co-authors were well aware that obtaining suitable devices and an internet connection may be prohibitively expensive for some potential participants. A budget smartphone or tablet will cost in the region of £100 to £200 (2020 UK prices), and a second-hand laptop around the upper end of that price bracket. Broadband connections start at around £25 per month, plus possible connection fees, and a SIM-only phone contract with 15GB data can be obtaining for around £15 per month. These costs may well be unfeasible for some participants, especially the initial outlay to obtain a device.

We discussed some practical solutions to prohibitive costs. Charities and some local authorities may be able to supply hardware to promote digital inclusion. Sharing a WiFi connection with a neighbour or borrowing a device from a family member or friend may be an option for some. One co-author highlighted the importance of ensuring participants are aware of the possibility of hidden costs when they are organising sessions. For instance, a typical hour-long Zoom call can use anything between 840MB and 2.5GB of data, so if a participant joined with their phone but forgot to turn on WiFi, they could incur an unexpected charge. Similarly, joining video calls by dialling in on a phone line may be charged at premium rates. Some devices may be set up to warn the user if they exceed a data allowance, but in other cases it could be a big shock. Facilitators can help by giving simple and clear instructions about how to avoid these costs where possible. Providers and funders need to compare the costs of remote interventions and face to face sessions in cost effectiveness studies.

### Summary of Current Practice

Previously we reported the details of online music making for people with dementia in terms of the types of sessions, platforms and activities being used (section Details). When these data were shared with the co-authors, their experiences were found to align with what we had described; in effect, they were using the same platforms and delivering sessions of the same types. This indicates that the experiences of the co-authors is broadly representative of the UK-wide situation based on the data. Subsequently, in section Discussion: Issues Affecting Viability of Online Singing we outlined the technical and accessibility challenges which co-authors had encountered, and explored some possible solutions. Again, many of these challenges were universally reported by co-authors, suggesting that even where practice has evolved separately, the same difficulties may prevent participants from joining in.

We are at an early stage in the diffusion of innovations in delivery of digital music making. The framing and context of a session can influence its success. For instance, some leaders found that care home residents struggled to focus attention on a TV screen or tablet computer where there were competing sensory stimuli in the room. There was agreement that the involvement of care home staff to engage participants in the session is crucial to its uptake—although this was of course very difficult during the height of the pandemic when many care homes were severely overstretched and short-staffed. Additionally, in care homes, the task of setting up technology often fell to one member of staff, and if this person was not available the session was missed. This highlights the need to build capacity for online interventions amongst care personnel.

For a minority of people with dementia, it appears that online singing created confusion. We discussed ways to improve in-session accessibility. For some, seeing lots of faces on the screen in a Zoom call was overwhelming. A suggestion in this case is to use “Speaker View” rather than “Gallery View.” For certain participants in residential care during lockdown, relatives wanted to join the session remotely. However, when a person with dementia did not understand why their loved one was appearing on a screen, this could disrupt the session. Co-authors who were running live online sessions frequently used the screen-sharing facility to display lyrics to songs, but it was also common to provide copies for printing for those who preferred to read the lyrics in this way. Facilitators also highlighted the importance of checking whether session participants had any other medical conditions which might affect their ability to engage with the technology.

Online music making could have potential advantages over in-person sessions. One contributor reported that some people who would not have had the confidence to attend a singing group in person have felt more comfortable attending digitally. Trying a group online gave them a taste of what it might be like in person; it felt less intimidating and they could leave without drawing attention to themselves if they were not enjoying it. This experience may encourage them to attend a group in person, post-COVID. However, one aspect of online sessions is that facilitators reported they are more intense and tiring than face-to-face sessions, so needed to be shorter. This consideration led to the inclusion of recommendations on sustainable practice and self-care; and, relatedly, to ensure that providers check their liability insurance covers them for online work.

In practice, only a minority of interventions take place one to one. Most facilitators will be interested in maximising the number of people who are able to access the sessions, although limits may be desirable in certain circumstances. The following recommendations therefore condense our findings with regard to successful provision of group singing sessions for people with dementia and their carers. They relate to selecting and preparing the most suitable technology, how to monitor the quality of the intervention and how to ensure the wellbeing of the participants, and have been formulated primarily with live videoconferenced sessions in mind.

### Limitations

There are several limitations to this community case study. The methods used to search for relevant interventions were not exhaustive and it is possible that some were missed. However, the use of a well-publicised nationwide database of musical activities means that the interventions listed are likely to be representative of the general picture. A further limitation is that the study focused on the UK, since all the contributors were UK based and the study was conducted to inform the implementation of interventional research which would take place in the UK. This may limit the applicability of the findings to non-UK contexts. A number of different types of activity have been trialled, and there is no way to estimate how far each has reached into the population of people with dementia and their carers. Although the paper mentions financial barriers to accessing the required technology, we did not consider the dual issue of whether participants pay for sessions, and how facilitators get paid (although we note that many of the pre-recorded video resources are freely available). Finally, the study only examined virtual and remote music-making approaches which had arisen for people with dementia as a result of COVID-19. Existing technologies which could potentially be used for this purpose, such as network mediated music-making, were not considered.

## Conclusions

It will be some time before we have an accurate picture of how people with dementia have been affected by the Covid-19 pandemic. Until a widespread programme of vaccination is implemented, it is likely that restrictions on group gatherings and shielding recommendations will continue. In the face of these challenges, the creativity and resilience demonstrated by arts organisations and practitioners as well as by people with dementia and their carers is heartening and inspiring. The digital developments of the pandemic may have wide-reaching implications for practice in a post-pandemic world, as their advantages become recognised. Virtual sessions may be attended by those who would not previously have the opportunity: people who live in geographically remote locations, those underserved by public transport, carers with multiple caring responsibilities, and people who have physical disabilities or mental health conditions which make it difficult to travel may all prefer to attend an online session. Furthermore, online sessions enable geographically separate groups and individuals to log on and spend time together. In addition, motivation to discover and use new technology for music making may have a positive carry-over effect to other areas of life. We hope that the recommendations outlined here will be relevant and helpful for musicians and dementia care providers to adapt to practice during the pandemic and beyond.

### Recommendations for Best Practice With Groups

**Getting to grips with technology: Making your intervention as widely accessible as possible**.

1. Choose platforms for sessions which are familiar and easy to use (run in web browser where possible) and offer alternative joining possibilities (e.g., dialling in).
2. Consider making sessions available on more than one platform (e.g., Zoom sessions and YouTube videos).
3. Think about providing offline alternatives for people with no internet access.
4. Give clear, specific and simple instructions about how to join or access the sessions. Follow guidelines about dementia-friendly communication when you are writing instructions for joining the sessions (e.g., The Dementia Engagement Empowerment Project, 2013).
5. Make participants aware of possible hidden charges and how to avoid or minimise these.
6. Have a designated volunteer or co-facilitator whose role is to provide technical support by telephone to participants, both prior to and during sessions if required.

**Wellbeing and safety: Managing risks and looking after participants**

7. Be careful when sending joining details not to reveal people's personal identifying information.
8. Think about how to protect participants' digital privacy (e.g., advise them to remove identifying features from the background or to use a digital backdrop, or changing the video to display first names only).
9. Check whether participants have any medical conditions which may affect their ability to use the technology and take appropriate mitigating actions where possible (e.g., epilepsy could be triggered by multiple screens on Zoom).
10. During the session, it may be productive to explore creative ways for participants to check in and convey their mood and energy levels.
11. If you have a co-facilitator or volunteers, consider designating someone who can take any participants who are distressed into a breakout room or phone them.
12. If the platform supports this, let participants know that they can type messages to you privately if they do not want to say something out loud.

**Quality control: Optimising the musical experience for participants**

13. Choose your session format based on the capabilities of your technology (e.g., is your internet connection fast and stable enough to support a live video conferenced session?)
14. Think about what accompaniment or backing will be used, if any. It may need to be recorded in advance.
15. If song lyrics are needed, consider how participants will access them. When sharing words on a screen the typeface needs to be large and clear.
16. Use the best equipment available; the facilitator's image and sound quality are vitally important. A large screen permits the leader to see more participants without scrolling.
17. The facilitator may wish to consider obtaining equipment such as a USB microphone or an audio mixer to improve the quality of their audio. Headphones help to reduce feedback.
18. Consider doing a test-run of audio set-up with a friend or colleague.
19. Understand how to mute participants individually and as a group.
20. The facilitator should check regularly with participants that they can hear the audio and that the balance between voice and accompaniment is suitable.

**Self-preservation: Helping practitioners to deliver digital music interventions sustainably**

21. Practitioners should check that any liability insurance they hold includes suitable cover for online sessions.
22. Online delivery is intense and unremitting. Sessions should be shorter than face to face practice, with time to recover in between online sessions. A co-facilitator is desirable, particularly with larger numbers of participants.
23. You may find it helpful to debrief after each session, either with a co-facilitator or another suitable person. Some artists also pay for individual supervision.

## Data Availability Statement

The original contributions presented in the study are included in the article/supplementary material, further inquiries can be directed to the corresponding author/s.

## Author Contributions

BD and JS conceived the design of this research, planned the procedure, carried out data collection consisting of consulting with practitioners and organisations, and drafted and revised the manuscript. BD performed searches to document online music interventions. RA, JB, CB, NC, ED, MH, IL, GJ, GM, AO'N, DN, GN, FP, PQ, AW, and CW contributed to knowledge about current practice either by reading an early draft of this paper and supplying comments, or in one-to-one conversation with BD, or during group discussion of the recommendations by videoconference. All authors have read and approved the final version of this paper.

## Conflict of Interest

DN is employed by Noble & Noble Limited, and works as a consultant with Live Music Now. The remaining authors declare that the research was conducted in the absence of any commercial or financial relationships that could be construed as a potential conflict of interest.

## Footnotes

^1^AbilityNet supports people of any age, living with any disability or impairment to use technology to achieve their goals at home, at work and in education.
